# Prevention of Adolescent Pregnancy in Anglophone Sub-Saharan Africa: A Scoping Review of National Policies

**DOI:** 10.34172/ijhpm.2020.185

**Published:** 2020-10-05

**Authors:** Bright Opoku Ahinkorah, Melissa Kang, Lin Perry, Fiona Brooks

**Affiliations:** ^1^School of Public Health, University of Technology Sydney, Ultimo, NSW, Australia.; ^2^Faculty of Health, University of Technology Sydney, Ultimo, NSW, Australia.

**Keywords:** Policies, Adolescent Pregnancy, Anglophone Sub-Saharan Africa, Scoping Review

## Abstract

**Background: **Despite the existence of preventive policies across sub-Saharan Africa, countries within the sub-region lead global rankings for rates of adolescent pregnancy. The aim of this scoping review was to identify and review national policies on the prevention of adolescent pregnancy in Anglophone sub-Saharan Africa.

**Methods:** Relevant policies were identified from searches of national government websites and the search engine Google. Recognised screening and data extraction processes were used; data were subjected to content analysis using a published Framework for Evaluating Program and Policy Design on Adolescent Reproductive Health. The Preferred Reporting Items for Systematic Reviews and Meta-Analyses (PRISMA) extension for scoping reviews guidelines was used in reporting the review.

**Results:**In line with the inclusion criteria that guided the selection of relevant policies in this study, 17 of 75 national policies were suitable for the analysis. All were backed by political recognition, were government and public initiatives, acknowledged a range of determinants of adolescent pregnancy and allocated human resources to policy activities. Few specified financial resourcing. Most policies acknowledged the importance of coordination and collaboration among public and private actors. All policies had objectives that addressed adolescent pregnancy but none were measurable or included timeframes. Provision of comprehensive sexuality education and adolescent reproductive health services were the most common recommendations. Monitoring and evaluation plans were present in all the policies. However, youth involvement in policy formulation, and plans for implementation, monitoring and evaluation was scarce.

**Conclusion:** Overall, national policy strengths were seen in relation to their political recognition, and all aspects of policy formulation. Policy implementation strengths and weaknesses were identified, the latter in relation to clear descriptions of financial resources. Importantly, the absence of measurable and time-bound objectives or formal evaluation of policy effectiveness confounds demonstration of what has been delivered and achieved. Youth involvement was notably absent in many policies. For future policy-setting, governments and policy-makers should make efforts to engage young people in policy development and to be transparent, realistic and address the necessary financial resourcing. They should set quantifiable policy objectives that provide a basis for assessing the adoption, uptake and effectiveness of policies in relation to measurable objectives.

## Background

 The 1994 International Conference on Population and Development was a landmark event for adolescent sexual and reproductive health (ASRH), as the ensuing Programme of Action acknowledged the need to explicitly address the sexual and reproductive health of young people, including adolescents.^1^ In line with this, improvement in ASRH became a global health priority.^2^ Subsequent decades have seen strategies adopted by international bodies such as the United Nations, World Health Organization (WHO), United Nations Children’s Fund (UNICEF) and United Nations Population Fund (UNFPA).

 Adolescence is the transitional period from childhood to adulthood, often characterized by physical, psychological and social changes.^3^ These changes make adolescence a distinctive period in the life-course in its own right, as well as an important time for laying the foundations of good health in adulthood.^4^ This period has been defined by WHO^5^ as the period from 10-19 years of age and is generally classified into early adolescence (10-14 years) and late adolescence (15-19 years).^3,6^

 The WHO guidelines ‘Preventing early pregnancy and poor reproductive outcomes among adolescents in developing countries’ recognized a number of interventions to prevent early pregnancy. Legal reform, and strategies to reduce child marriage, increase contraceptive use, reduce coerced sex and unsafe abortion and increase the use of maternity healthcare services were highlighted.^7^ Target 5.3 of the 17 Sustainable Development Goals (2016–2030) articulates specific aims towards addressing adolescent pregnancy, and includes the elimination of harmful practices such as child, early and forced marriage.^8^ Despite these efforts, adolescent pregnancy remains a major challenge in developing countries.^9-11^ Many countries within sub-Saharan Africa lead global rankings for rates of adolescent pregnancy; for example, reported births per 100 000 teenage women are: in Niger, 203.6; Mali, 175.4; Angola, 166.6; Mozambique, 142.5, and Guinea, 141.7.^12^

 Early and unintended pregnancies among adolescents are associated with adverse health, educational, social and economic outcomes.^11^ Adolescents may be biologically immature with incomplete pelvic growth. Adolescent pregnancies are at greater risk of eclampsia and post-partum complications such as haemorrhage,^13^ and pregnancy-related conditions are the second major cause of death among adolescent girls in developing countries.^14^ The health implications of adolescent pregnancy also extend to the health of their infants, with studies demonstrating higher rates of perinatal death and low birth weight among babies born to mothers under 20 years of age.^15,16^ Infants born to adolescents are also at risk of malnutrition, low and delayed mental and physical development, poor parent-child attachment (degree of closeness/warmth experienced in the relationship between child and parent) and less education.^17^ Pregnant adolescents can develop psychological problems from social stigma, and suffer physical and domestic violence.^18^ Adolescent pregnancy also disrupts young women’s schooling and endangers their future economic opportunities, including reducing job market opportunities,^19^ and can initiate a poverty cycle in their families.^20^

 In line with international recommendations for addressing adolescent pregnancy, several countries in sub-Saharan Africa have made efforts to develop and implement such policies.^18,20,21^ To assess the role, impact and/ or outcome of national policy in reducing adolescent pregnancy, it is useful to first understand the extent and scope of national policy in the region, and then to examine empirical data over the term of policy and beyond. This review therefore aims to scope and review the policies relevant to prevention of adolescent pregnancy in the 24 Anglophone sub-Saharan Africa countries.

## Methods

 The authors used the methodological framework presented by Arksey and O’Malley^22^ for this scoping review, which included five key phases: (1) identifying the research question, (2) identifying relevant studies, (3) study selection, (4) charting the data, and (5) collating, summarizing, and reporting the results. The optional ‘consultation exercise’ of the framework was not conducted.

###  Phase 1: The Research Question

 This review was guided by the two research questions and objectives outlined below.

####  Review Questions

1. What policies in Anglophone sub-Saharan Africa are relevant to prevention of adolescent pregnancy?
2. To what extent do these policies describe their stages of development and implementation and the key factors and actors required during these stages?

**Figure 1:**
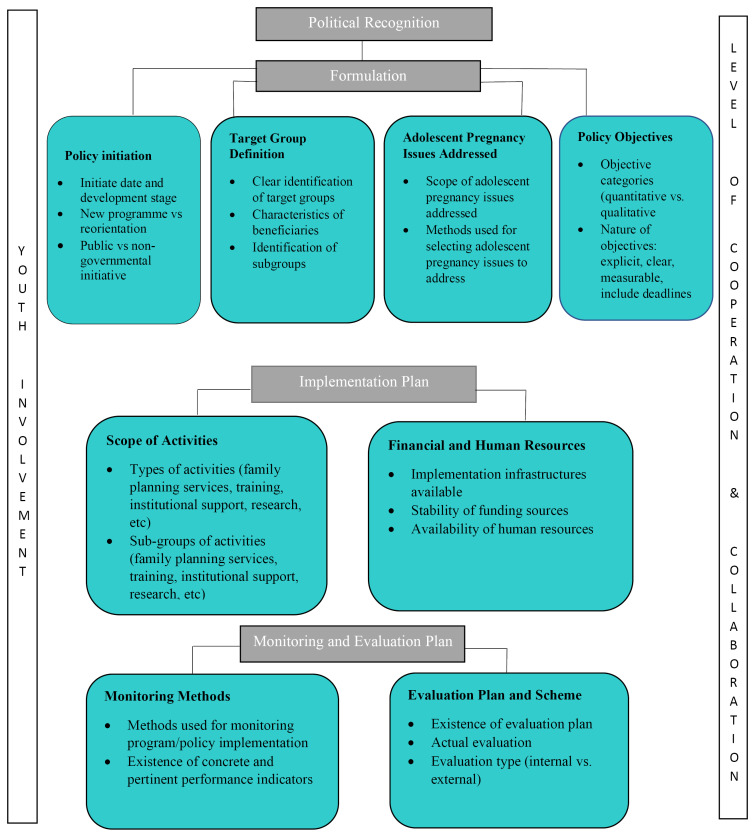
Reproduced With Permission: Conceptual Framework for Evaluating Program and Policy Design on Adolescent Reproductive Health. Source: Calves.^24^

To develop our objectives and guide the analysis, we adapted the conceptual Framework for Evaluating Program and Policy Design on Adolescent Reproductive Health described by Calves^23^ (Figure 1). This framework was chosen because it focuses on the assessment of the first phases of policy-making processes: political recognition, policy formulation, the implementation plan, and the monitoring and evaluation plan. It also considers implementation issues: the level of coordination, collaboration and youth involvement in adolescent reproductive health policy and program design.^23^ Thus, it was seen as suitable to frame the response to the review questions, and was applied in this review as follows. Political recognition refers to the extent to which adolescent pregnancy considerations are acknowledged as national issues and priorities. This could be seen in statements by political leaders indicating their support for prevention of adolescent pregnancy. The formulation component focuses on the date of creation and stage of development of the policy; whether a policy is new or a reorientation of an existing policy, national/international or governmental/nongovernmental initiative; the definition of the target group, the pregnancy issues addressed and policy objectives. The implementation plan considers the scope of activities, financial and human resources employed to reduce adolescent pregnancy. The monitoring and evaluation plan focuses on the monitoring methods, and the existence of an evaluation plan within the policies. In terms of the level of coordination and collaboration, the roles as well as the number of partners involved in public and private efforts are examined, with the level of coordination among policy actors regarded as an index of likely success. Finally, the level and nature of youth involvement at each stage of the policy design process is considered.^23^

 The scoping review objectives were to:

1. Identify contemporary policies relevant to prevention of adolescent pregnancy in Anglophone sub-Saharan Africa,
2. Examine the role of political recognition in the development of these policies,
3. Assess the role of policy initiation, target-group definition, adolescent reproductive health issues, and policy objectives in the formulation of these policies,
4. Identify and describe policy implementation plans,
5. Identify policy monitoring and evaluation plans, and any evidence of their effectiveness,
6. Assess the extent of cooperation and collaboration in the development of these policies and,
7. Explore the level of youth involvement in the development of these policies.

###  Phase 2: Identifying Relevant Policies

 Data for the study were obtained from policies on the prevention of adolescent pregnancy in Anglophone sub-Saharan Africa. Inclusion criteria were that policies:

- Were national government policies,
- Were published between 2010 and 2019,
- Targeted adolescents or youth, and
- Included strategies or interventions aimed at reducing adolescent pregnancy.

A preliminary search was conducted in Scopus, Medline/PubMed, CINAHL/EBSCO and Web of Science using search terms such as teenage pregnancy policies, school health policies, health policies, adolescent health policies, maternal and child health policies, sexual and reproductive health policies, health promotion policies, health education policies, health services policies or youth policies, and Anglophone countries in sub-Saharan Africa. This search yielded no results. The search therefore focused on a manual search of websites of the national health departments/ ministries and the most used search engine, Google.^24^

####  Manual Search of Websites of National Health Departments

 The search took place between February 11, 2019 and March 9, 2019. The 24 Anglophone sub-Saharan African countries’ national health department websites were searched manually. Table 1 shows a list of the countries and the websites from which relevant policies on adolescent pregnancy included in this study were found. Policies published in the last decade were considered for this review in order to have recent or current understanding of the policy measures. Given possible variations in Anglophone and Francophone political systems^25-27^ and policy processes^28-31^ and to limit the review to policies published in English, the search was limited to national policies developed by Anglophone countries.

**Table 1:** List of Countries and Sources of Data on Policies Relevant to Prevention of Adolescent Pregnancy

**Country**	**Website**
Botswana	https://www.moh.gov.bw
Burundi	https://ghdx.healthdata.org/organizations/ministry-public-health-burundi
Eritrea	https://ghdx.healthdata.org/organizations/ministry-health-eritrea
Ethiopia	https://www.moh.gov.et/ejcc/
Ghana	https://www.moh.gov.gh; https://www.mogcsp.gov.gh/
Kenya	https://www.health.go.ke; https://www.popcouncil.org/
Lesotho	https://www.gov.ls/ministry-of-health/
Liberia	https://www.moh.gov.lr
Malawi	https://www.health.gov.mw
Mauritius	https://www.health.govmu.org
Namibia	https://www.mhss.gov.na; https://www.moe.gov.na
Nigeria	https://www.health.gov.ng
Seychelles	https://www.health.gov.sc
Sierra Leone	https://www.health.gov.sl; https://www.afro.who.int
South Africa	https://www.health.gov.za
South Sudan	https://www.moh-rss.org/
Sudan	https://ghdx.healthdata.org/organizations/federal-ministry-health-sudan
eSwatini^a^	https://www.gov.sz/index.php/ministries-departments/ministry-of-health
Rwanda	https://www.moh.gov.rw
Tanzania	https://www.moh.go.tz
The Gambia	https://www.moh.gov.gm
Zambia	https://www.moh.gov.zm
Zimbabwe	https://www.mohcc.gov.zw
Uganda	https://www.health.go.ug

^a^eSwatini previously known as Swaziland.

####  Searching Using Google

 A search was conducted using the search engine Google with no date restrictions, with search terms focused on teenage pregnancy policies, school health policies, health policies, adolescent health policies, maternal and child health policies, sexual and reproductive health policies, health promotion policies, health education policies, health services policies and youth policies in Anglophone countries in sub-Saharan Africa.

 Next, a more specific search was conducted through Google using the same search terms but replacing ‘Anglophone countries in sub-Saharan Africa’ with the names of each of the 24 countries. The first 50 hits (as sorted by relevance by Google) were screened for relevant policies in line with recommendations of previous studies.^32,33^ The first 50 hits were the point beyond which no further relevant policies on adolescent pregnancy appeared. In total, 41 policies were first sourced using Google, and 34 policies were retrieved from the government websites.

###  Phase 3: Study Selection 

**Figure 2:**
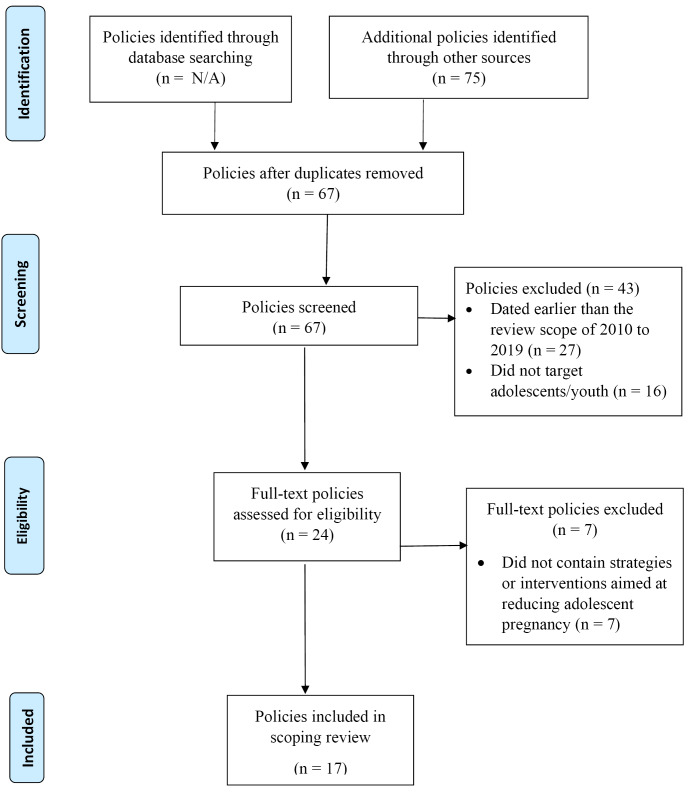
The Searching and Screening Process; An Adapted PRISMA Extension for Scoping Reviews Flow Diagram.^35^ Abbreviation: PRISMA, Preferred Reporting Items for Systematic Reviews and Meta-Analyses.

 A three-stage screening process was used to determine the final sample of policies for analysis, as set out in the PRISMA (Preferred Reporting Items for Systematic Reviews and Meta-Analyses) extension for scoping reviews flow diagram.^34^ In the first and second stages, duplicate records and then policies published before 2010 or that did not target adolescents/youth were removed. The remaining 24 policies were read in full, seeking evidence of strategies or interventions aimed at reducing adolescent pregnancy. A further seven were excluded, leaving 17 eligible policies from 12 of the 24 countries. The policy search and screening process is shown in Figure 2.

###  Phase 4: Charting the Data

 Using NVivo version 11, a matrix was created for the extraction and display of data from the policies guided by the ‘Conceptual Framework For Evaluating Program And Policy Design On Adolescent Reproductive Health’ (Figure 1).^24^ The matrix assisted with data organisation by ensuring that extracted data were mapped according to the components of the conceptual framework. First, all policy documents were imported into NVivo. Secondly, ten ‘Nodes’ were created which corresponded to the ten framework components: (1) political recognition, (2) policy initiation, (3) target group definition, (4) adolescent pregnancy issues addressed, (5) policy objectives, (6) scope of activities, (7) financial and human resources, (8) monitoring and evaluation plan (9) level of cooperation and collaboration and (10) level of youth involvement. Data charting was an iterative process in which two authors extracted data and updated the data charting form as relevant content emerged.

###  Phase 5: Collating, Summarizing, and Reporting the Results

 Content analysis^35^ was applied by the first author to analyse the policies and guided by the ‘Conceptual Framework For Evaluating Program and Policy Design on Adolescent Reproductive Health’ (Figure 1). Content analysis involves a systematic coding and categorizing approach used for exploring large amounts of textual information to determine trends and patterns of words used, their frequency, their relationships, and the structures and discourses of communication.^36^ The PRISMA extension for scoping reviews guidelines was used in reporting the review.^34^

## Results

 The key components of the policies relevant to prevention of adolescent pregnancy in Anglophone sub-Saharan Africa based on the ten key elements of the Framework (Figure 1) are presented in Table 2.

**Table 2:** Summary of Policies Relevant to Prevention of Adolescent Pregnancy in Anglophone Sub-Saharan Africa

**Policy Title (Country, Date)**	**Political Recognition**	**Policy Initiation**	**Target Group Definition**	**Adolescent Pregnancy Issues/Determinants Addressed**	**Policy Objectives**		**Scope of Activities**	**Resources**	**M & E Plan**	**Cooperation and Collaboration **	**Youth Involvement**
					**Quantitative vs. Qualitative**	**Explicit, Clear, Measurable, Deadlines**					
Revised National Youth Policy (Botswana, 2010)^41^	P	Revised	Youth (15-35 y) Additional subgroups of youth	Poverty, Unemployment	Qualitative	X	IEC (peer education, youth life skills, youth and health empowerment programmes), health services (youth friendliness, include vulnerable groups), institutional support (supportive legal environments)	Financial X Human P	P	P	X
National Gender Policy (Ghana, 2015)^44^	P	New	Boys and girls	Poverty, Sexual violence	Qualitative	X	IEC (education, school retention programmes), institutional support (welfare department, trafficking secretariat, human rights court)	FinancialP Human P	P	P	X
Adolescent Health Service Policy and Strategy (Ghana, 2016)^39^	X	New	Young people (10-24 y)	Access to family planning services, coerced sex, concurrent partners, child marriage, contraceptive use, early sexual initiation, multiple sexual partners, sexual violence	Qualitative	X	IEC (Social and behavioural change communication strategy), health services (increase access for adolescents), training (needs assessment of staff, and capacity building of staff)	Financial P Human P	X	P	P
National Adolescent Sexual and Reproductive Health Policy (Kenya, 2015)^42^	P	Revised	Adolescents (10-19 y) Additional subgroups of adolescent	Availability of SRH services, child marriage, coerced sex/sexual abuse, early sexual initiation, low self-confidence, multiple sexual partners, poverty	Qualitative	X	IEC (parents, communities, adolescents, professionals, CSE, digital platforms to access information), health services (strengthen capacities to provide appropriate information and services), training (build the capacity of healthcare providers, institutional support (ensure attainment of ASRH rights), research (data management and analysis)	Financial X Human P	P	P	P
National Youth Policy (Malawi, 2013)^43^	P	Revised	Youth (10-35 y)	Unemployment, early marriage	Qualitative	X	IEC (youth involvement in program design, CSE, target school drop outs, vulnerable youth), health services (adequate and accessible youth friendly health services), institutional support (advocate for increase in the legal age of marriage, regulations and enforcement of laws that advance youth reproductive health including sexual violence)	Financial X Human P	P	P	P
National Health Policy Framework (Namibia, 2010)^53^	P	New	Young people and adolescent	Low contraceptive prevalence rate Unmet need for family planning	Qualitative	X	IEC (community, adolescents), health services (adolescent-friendly), training (staff of health services)	Financial P Human P	P	P	X
Education Sector Policy for the Prevention and Management of Learner Pregnancy (Namibia, 2010)^39^	P	New	Learners of school-going age	Early sexual debut, forced sex, gender inequity	Qualitative	X	IEC (CSE), health services (counselling)	Financial P Human P	P	P	X
Reproductive Health Policy (Seychelles, 2012)^51^	P	New	Adolescents Youth	Early sexual debut, sexual abuse, sexual violence, unprotected sex, intergenerational sex	Qualitative	X	Health Services (access to ASRH), Training (teachers, counsellors, professionals), Institutional support, (school health programme)	Financial X Human P	P	P	X
Reproductive, Maternal, Newborn, Child & Adolescent Health Policy (Sierra Leone, 2017)^40^	P	New	Adolescents		Qualitative	X	IEC (CSE, adolescents ), health services (adolescent friendly, increase uptake), training (health workers), institutional support (address legal and sociocultural barriers to health services, advocate elimination of harmful practices)	Financial X Human P	P	P	X
Integrated School Health Policy (South Africa, 2012)^48^	P	New	Youth	Low contraceptive use Early sexual debut	Qualitative	X	IEC (CSE)	Financial X Human P	P	P	P
National Adolescent and Youth Health Policy (South Africa, 2017)^47^	P	New	Adolescent and youth (10-24 y)	Lack of access to SRH services	Qualitative	X	IEC (CSE) Health services (access to youth friendly services)	Financial P Human P	P	P	X
National Family Planning Policy (South Sudan, 2012)^54^	P	New	Adolescents Youth	Illiteracy, early marriage, access to contraceptives, spacing or limiting childbirth	Qualitative	X	IEC (adolescents) Health services (provision of accessible services)	Financial P Human P	P	P	X
National Reproductive Health Policy (South Sudan, 2013)^52^	P	New	Adolescents	Inadequate access to SRH services, early marriage, gender-based violence	Qualitative	X	Health services (increase availability, ensure equity of access, improve facilities), Institutional support (eradicate gender-based discrimination and violence)	Financial P Human P	P	P	X
National Policy on Sexual and Reproductive Health (eSwatini, 2013)^49^	P	New	Youth Adolescents	Inadequate access to SRH services	Qualitative	X	IEC (schools, community), health services (enable resources)	Financial P Human P	P	P	X
The National Health Policy (Tanzania, 2017)^55^	P	Revised	Adolescents	Low supply of family planning methods, limited knowledge	Qualitative	X	Health services (ensure quality services to adolescents, strengthen services), institutional support (ensure law enforcement re gender-based violence)	Financial X Human P	P	P	X
Gender and Women Empowerment Policy 2010-2020 (The Gambia, 2010)^45^	P	New	Boys and girls	Early marriage, limited access to SRH information	Qualitative	X	IEC (schools, out of school, community)	Financial X Human P	P	P	X
Gambia National Gender Policy 2010-2020 (The Gambia, 2010)^46^	P	New	Boys and girls	Early sexual debut; school drop-out	Qualitative	X	IEC (schools, community), institutional support (school retention, for reporting sexual abuse)	Financial X Human P	P	P	X

Abbreviations: ASRH, adolescent sexual and reproductive health; CSE, comprehensive sexuality education; IEC, information, education and communication; M & E, monitoring and evaluation; SRH, sexual and reproductive health. X: Not described, P: Described.

###  Political Recognition

 All the policies except the Adolescent Health Service Policy and Strategy of Ghana^37^ covered issues on adolescent reproductive health and had statements by political leaders indicative of political recognition. Some of these statements reflected the efforts expended in this area, and included statements such as ‘significant efforts have been made by the government in collaboration with the community and stakeholders in expanding, improving and distribution of the reproductive, maternal, newborn, child and adolescent health services to the target population.’^38^ Such statements by political leaders reflect their will to improve ASRH through collaboration with other major stakeholders. Aspirational statements were common in most of the policies. For example, policies of Namibia and Sierra Leone had statements such as, ‘as we move closer towards the 2015 Millennium Development Goals and Vision 2030, it is time to implement a new policy to address adolescent pregnancy that will make a real and sustainable difference to the lives of children in Namibia’^39^ and that, ‘the health and well-being of women, mothers, newborns, children and adolescents is a priority for the Government of Sierra Leone.’^40^

###  Policy Initiation

 The 17 policies included in the scoping review were initiated between 2010 and 2017. Four were revisions^38,41-43^ and the remainder were new policies. Whilst the new policies were initiated to improve the health and well-being of the general population of those countries, especially adolescents, the revised policies were initiated to meet the changing health needs of the general population, including adolescents. For instance, Kenya initiated a National Adolescent Sexual and Reproductive Health Policy in 2015 based on the idea that ‘many continuing and emerging issues have come to the fore as a result of advances in information, communication and technology and the resultant exposure to materials and practices that influence young people’s behaviour.’^42^ Similarly, Malawi initiated a Revised National Youth Policy in 2013 ‘to embrace new challenges and other emerging issues currently facing the youth in Malawi.’^43^

###  Target Group Definition

 We examined whether the policies identified adolescents/youths as the target population, provided clear characteristics of the expected beneficiaries and mentioned sub-groups of adolescents/youth. Thirteen policies specifically mentioned adolescents/youth as the target group, whilst the remaining three used terms including ‘boys and girls’^44-46^ and ‘learners of school-going age.’^39^ Where the focus was on adolescents, most policies defined this as persons aged 10-19 years. However, the definition of youth varied from one policy to another. For instance, in the National Adolescent and Youth Health Policy of South Africa^47^ and the Adolescent Health Service Policy and Strategy of Ghana,^37^ ‘youth’ was defined as persons aged 10-24 years, but as persons aged 10-35 years and 15-35 years in the National Youth Policy of Malawi^43^ and the Revised National Youth Policy of Botswana,^41^ respectively.

 Apart from identifying adolescents/youths as the primary target group, the National Adolescent Sexual and Reproductive Health Policy of Kenya^42^ and the Revised National Youth Policy of Botswana^41^ went a step further to identify sub-groups of adolescents/youths (see Table 2). Other groups such as parents, community leaders, teachers and health providers were considered ‘secondary targets’ of specific training; information, education and communication or advocacy activities.^48^

###  Adolescent Pregnancy Issues Addressed

 This aspect of the assessment looked at the adolescent pregnancy issues addressed, taking into consideration the specific determinants of adolescent pregnancy covered in the policies. Child marriage, gender-based violence, early sexual initiation, multiple sexual partners, coerced sex, lack of contraceptive education, lack of affordable and adequate contraceptive commodities and inconsistent and incorrect condom use, poverty, illiteracy, unemployment and school drop-outs were referred to as the determinants of adolescent pregnancy in the policies. Whilst some of the policies referred to all of these determinants, others referred to only a limited number. For instance, the Gender and Women Empowerment Policy of the Gambia^45^ describes the determinants of adolescent pregnancy as follows: ‘The girl-child has limited understanding of the basic physiology of the menstrual cycle, poor sexual relationship, and limited knowledge on the causes of pregnancy and that one act of sexual intercourse can lead to pregnancy. This can be attributed to the fact that most adolescent and youth have very limited access to sexual and reproductive health information. This is because sexuality issues are shrouded in taboos and parents do not talk to their children about sex and sexuality issues; as a result they get their information from their peers who are also not adequately informed.’ Furthermore, the National Policy on Sexual and Reproductive Health of eSwatini^49^ also referred to the determinants of adolescent pregnancy as follows: ‘Adolescents and youth in Swaziland do not have adequate information and accessibility to services which will enable them to make informed decisions on their sexuality and reproductive health.’

 Adolescent pregnancy issues were identified from a range of sources including primary data, secondary data, informal feedback from the field and international standards. However, secondary data analysis was the dominant method used and in most instances, the Demographic and Health Survey (DHS) was the major secondary data source. The DHS is a nationwide survey collected every five-year period across low- and middle-income countries.^50^

###  Policy Objectives

 All 17 policies set both general and specific objectives. All objectives were qualitative in the sense that they were not expressed in quantifiable terms and were not measurable. Examples of qualitative objectives were to, ‘Improve access to information on health and health services relevant to the age and gender speciﬁc needs of adolescents and young people to enable them make informed decisions.’^37^ and ‘Address the special *sexual and reproductive health and rights*-related needs of marginalized and vulnerable adolescents’^42^ It must however be acknowledged that although the National Adolescent Sexual and Reproductive Health Policy of Ghana stated qualitative objectives, a portion of the policy had some quantitative indicators as benchmarks for evaluating the objectives. These benchmarks included ‘Increasing condom use at sexual debut for 15-24 years olds from 67% in 2010 to 75% in 2020 among women and 58% in 2010 to 65% in 2020 among men,’ and to increase the age of sexual debut among 12-14 years olds from 10 years in 2015 to 15 years in 2020.^42^The objectives of the policies differed in terms of explicitness (stating desired results rather than referring to activities to be performed), clarity (well-defined terms and concepts), measurement (whether objectives allow for verification of achievement) and timeframes (whether specific dates have been established for reaching each objective).

 Some of the objectives in the policies were explicit and clear on prevention of adolescent pregnancy, for example ‘To increase learner education about sexual responsibility and sexual health to help prevent learner pregnancies,’^39^ and to, ‘Strengthen access to adolescents-friendly sexual and reproductive health information, counselling and medical care services for various groups of adolescents.’^51^ However, others were broad and did not provide direct solutions to adolescent pregnancy prevention. Most such objectives were in the National Gender Policy of Ghana and the Integrated School Health Policy of South Africa. Examples were ‘To improve women’s economic opportunities including engendering macro-economic and trade policies so that the basic and strategic needs of both men and women are addressed’^44^and *‘*To provide preventive and promotive services that address the health needs of school-going children and youth with regard to both their immediate and future health.’^48^ Only the Gambia National Gender Policy had an objective that included a timeframe: to ‘Enact laws that will prohibit all forms of gender-based violence by 2020.’^46^

###  Scope of Activities

 All the policies outlined a broad scope of activities to implement their objectives. The activities included information, education and communication; advocacy; provision of adolescent reproductive health services; comprehensive sexuality education; youth empowerment; the training of healthcare providers, parents and community members and political and legal actions that support adolescent reproductive health. Of all these activities, comprehensive sexuality education and provision of adolescent reproductive health services were the most common (see Table 2). In terms of recency, comprehensive sexuality education was mentioned mostly in policies that were published between 2013 and 2017 (see Table 2).

###  Financial and Human Resources

 Human and financial resources are important in the development of policies. All the policies identified the human resources necessary to implement the activities outlined. The human resources included ministries, health professionals, local government authorities, educators, development partners, civil society organisations and non-governmental organisations. Eight of the policies specifically mentioned financial resources and sources of funding. For instance, the National Reproductive Health Policy of South Sudan stated that ‘the government of South Sudan is providing the bulk of financial resources for reproductive health, with the support of various bilateral and multilateral development partners, including UN agencies.’^52^ Similarly, the National Policy on Sexual and Reproductive Health of Swaziland mentions that ‘financing implementation of this policy shall be funded primarily by Government with contributions from development partners and private sector.’^49^

###  Monitoring and Evaluation Plan 

 The review considered whether the policies specified or described a monitoring and evaluation plan and performance indicators; if so, whether performance indicators were concrete and pertinent in relation to the objectives of the policies. All the policies had plans for monitoring and evaluation except the Adolescent Health Service Policy and Strategy of Ghana.^37^ Most included general guidelines for monitoring and evaluation, but the content on monitoring and evaluation in some of the policies was brief. For instance, the Reproductive Health Policy of Seychelles provided guidelines but little discussion of monitoring and evaluation needs, recommending, ‘Monitoring and evaluation will be done through: conducting routine surveys and tracking inter-mediate and long term indicators based on programme objectives and national Inter-Reproductive Health Programme.’^51^ Few policies included performance indicators, and most of the indicators were neither concrete nor pertinent to the objectives of the policies. For instance, performance indicators such as ‘coverage of services,’ ‘quality of services’ and ‘sustainability of school health services in all districts’^48^ were not defined in measurable ways. The most frequently cited monitoring methods were periodic surveys, reports and meetings (see Table 2).

###  Level of Coordination and Collaboration

 The level of coordination and collaboration was assessed by examining the roles as well as the number of partners involved in public and private efforts. Apart from listing non-governmental organisations and national and international agencies as the main participants in policy implementation, all policies acknowledged the importance of coordination and collaboration. While all the policies generally stressed the need for collaboration between government and non-governmental actors, only one made specific reference to such coordination in its policy objectives as follows: ‘Promote partnership and inter-sectoral collaboration among adolescent and youth groups, relevant institutions and communities in the provision and utilization of Adolescent and Youth Responsive Health Service.’^39^

###  Level of Youth Involvement

 The level and nature of youth involvement were examined at each stage of the policy design process (formulation, implementation plan, monitoring and evaluation), distinguishing between direct and indirect modes of involvement. Examples of indirect youth involvement included surveys with adolescents, adolescent focus groups and informal feedback from adolescents from the field. Direct involvement refers to activities in which adolescents are collaborative partners.^23^ In terms of indirect youth involvement, the kinds of data used to develop the policies implied that youths were involved in surveys, focus groups and other means of data collection. Few of the policies talked about direct youth involvement, where adolescents/youth were involved as collaborative partners, actively involved in activities for the formulation, implementation plan, monitoring and evaluation of the policies (see Table 2).

## Discussion

 This scoping review identified 17 current policies relevant to the prevention of adolescent pregnancy in Anglophone sub-Saharan Africa. All were backed by political recognition, and targeted adolescents/youth. All policies referred to or addressed a range of determinants of adolescent pregnancy. Although most of the policies’ objectives addressed adolescent pregnancy, none was measurable and only one included a timeframe. Comprehensive sexuality education and provision of adolescent reproductive health services were the most common recommendations across policies. All policies identified human resources to support policy activities but few had financial resources allocated. Most acknowledged the importance of coordination and collaboration among public and private actors. Monitoring and evaluation plans were present in all policies. However, youth involvement in policy formulation, plans for implementation, monitoring and evaluation was not adequately addressed.

 Political recognition was in evident in the development of these policies. This is in line with the findings of Dye^56^ and Stover and Johnston,^57^ who identified political recognition as a necessary first step in policy and program development, and Birdthistle and Vince-Whitman,^58^ who found that the existence of clear national guidelines was often pivotal to an ASRH program’s success. However, as all the policies were government and public initiatives, statements of governmental support were to be expected.

 A wide range of determinants of adolescent pregnancy was identified in the policies, as in other studies from sub-Saharan Africa.^18,21,59,60^ Such information is useful for countries to enable them to set comprehensive and systematic strategies and targets to address adolescent pregnancy. The policies had appropriate objectives, and comprehensive sexuality education was the predominant recommended activity. Developmentally appropriate, evidence-based education about human sexuality and sexual reproduction, provided over time by paediatricians, schools, other professionals and parents, is important to help children and adolescents make informed, positive, and safe choices about healthy relationships, responsible sexual activity, and their reproductive health.^61-63^ The enactment of such rational choices may depend on the accessibility and availability of sexual and reproductive health services for adolescents, and that they receive the needed support from policy-makers and funders. Adequate support for the provision of reproductive health services will enhance access and therefore use of the services by adolescents.

 The need for coordination and collaboration between public and private actors is a key policy component. Senderowitz^64^and Hughes and McCauley^65^ explained why coordination and collaboration between public and private actors is needed in policy development. They viewed collaboration and coordination of public and private efforts as essential elements of successful policy and program design, contributing to flexible programming. In relation to adolescent reproductive health policies, Calves^23^ explained that coordination and collaboration of governmental and nongovernmental efforts in adolescent reproductive health are critical, particularly because nongovernmental organisations often play a vital role in providing ASRH information and services. Despite the existence of human resources supporting policy activities, few policies considered financial resources a key element. In line with this finding, Calves^23^ explained that most government policies in Togo, Cameroon and Burkina Faso provide inadequate information on financial resources.

 The majority of the policies had plans for monitoring and evaluation, key elements in health policy. O’Neill et al,^66^ for example, explain that monitoring and evaluation are a necessary basis for accountability, to provide documentation of programs, demonstrate whether they are achieving their intended influence and indicate the extent to which they are reaching their target audience.^67^ Hence, ensuring that monitoring and evaluation elements are in place at program start-up has been considered important to help demonstrate policy and program success and to identify aspects of policies and programs that require formulating or strengthening.^23^

 The level of youth involvement in the policies was low. Only four of the policies mentioned youth involvement, and only in the forms of surveys with adolescents, adolescent focus groups and informal feedback from adolescents. There was no evidence of involvement of adolescents as collaborative partners in the implementation of policy activities or in monitoring and evaluation. The absence of youth involvement indicated the low priority given to the voice of youth in policy development, which hinders the ability of youth to contribute to policy effectiveness. Similarly, Calves^23^ found that few government policies identified youth involvement as intrinsic to policy formulation, planning for implementation, monitoring or evaluation despite the vital role attributed to youth involvement in all stages of youth policy and program development, implementation and evaluation.^64,65^ The minimal involvement of youth in policies relevant to prevention of adolescent pregnancy is likely to negatively affect the adoption, uptake and effectiveness of these policies. However, it is important to acknowledge that policy documents may not describe all processes and activities undertaken in the development of the policy including youth involvement.

 Whilst the policies had appropriate objectives, none were measurable or time-limited; points also noted by Calves.^23^ This challenges verification of policy achievements and time requirements, making it difficult to determine whether the policies are effective. None of the policies were accompanied by a formal process of evaluation of their effectiveness in meeting the primary objective of reducing adolescent pregnancy rates; however, an indirect approach to assess their efficacy has been adopted by a number of countries. These countries have analysed pregnancy rates before and after policy implementation using data from the DHS, which has been undertaken at regular intervals. Of the 12 countries whose policies were assessed, only three had data on adolescent pregnancy rates before and after the policies cited in this review (see Table 3). Although not representative of the countries, assessment of pregnancy rates before and after policy launch in these three countries provides at least an indication of policy effectiveness in Anglophone sub-Saharan Africa. However, there is the need to take into consideration that policies and laws are important but on their own are likely insufficient to drive change.

**Table 3:** Pre and post policy pregnancy rates of selected countries

**Country; date**	**DHS Survey; Years**	**Pre-policy** ^a^	**Post-policy** ^a^
Malawi; 2013	2010; 2015	25.6%	29.0%
Namibia; 2010	2006; 2013	15.4%	18.6%
South Africa; 2012	2003; 2016	11.9%	15.6%

Abbreviation: DHS, Demographic and Health Survey. ^a^Pre- and post-policy adolescent pregnancy rates.

 As shown in Table 3, pregnancy rates in Malawi increased from 25.6% to 29.0% after launch of their National Youth Policy that specifically aimed to reduce adolescent pregnancy. Similarly, despite the existence of the National Health Policy Framework of Namibia in 2010, adolescent pregnancy rates increased from 15.4% to 18.6%. Like Malawi and Namibia, South Africa also experienced a rise in pregnancy rates from 11.9% to 15.6% despite the existence of the Integrated School Health Policy. The rising pregnancy rates experienced in these countries may result from barriers such as requirements for parental/spousal consent to access ASRH services, stigma surrounding premarital sex, pressure from community members to prove and protect fertility after marriage and the inability of adolescents to access long-acting contraceptive methods and safe abortion care from health workers.^68^ To understand these barriers and how they inhibit the effective implementation of policies on adolescent pregnancy, it is important to systematically review how the policies were developed, implemented and evaluated, and to address the gaps in these policies, including inadequate financial resources, low youth involvement and non-measurable and time-limited objectives.

## Review Limitations

 Despite the use of a conceptual framework to assess the various components of the policies, this study could not take into account the multiple drivers of adolescent pregnancies. Also, the search conducted for this review was restricted to national policies in the English language found on government websites and via Google. It is possible that some national government policies, or separate policy implementation plans, may not be publicly available, or not available online, and may therefore have been missed. Again, using this approach to account for changes in adolescent pregnancy rates, given the wide range of socio-economic and cultural factors is a key limitation of the study. Inclusion of policies from non-English-speaking countries, multi-national and Non-governmental organizations such as the WHO may have changed the picture. We acknowledge that legislative differences might be one of the causes for differences in policies. Finally, we acknowledge that the policy documents may not describe all processes and activities undertaken in the development of the policy. More importantly, there is the need for sub-national policy documents given that some countries have devolved local governments.

## Conclusion and Implications

 This review contributes a broad perspective on policies in sub-Saharan Africa and their role in addressing adolescent pregnancy rates. Findings can serve as a benchmark for future revision of national policies geared towards addressing adolescent pregnancy in sub-Saharan Africa. Guided by the conceptual framework for evaluating program and policy design on adolescent reproductive health,^23^ we found that all national policies relevant to pregnancy prevention in Anglophone sub-Saharan Africa during the target years were backed by political recognition; were formulated for clearly defined target groups and referred to adolescent pregnancy issues with clear and explicit objectives. On the whole, strengths were seen in policy implementation, monitoring and evaluation plans which included clear descriptions of scope of activities, human resourcing, and collaboration and coordination between public and private actors. However, there were gaps in relation to financial resourcing and youth involvement. Importantly, the absence of measurable and time-bound objectives or formal evaluation of policy effectiveness confounds demonstration of what has been delivered and achieved. Further evaluation and more time for policy impact will be required to demonstrate whether and how policies may be achieving meaningful reductions in adolescent pregnancy rates. Further studies should seek to include sub-national policy documents where countries have devolved local governments.

 For future policy-setting, governments and policy-makers are encouraged to be transparent and realistic about the necessary financial resourcing and identify funding sources. Quantifiable policy objectives should be set to provide a basis for assessing the implementation and outcomes of policies: their adoption, uptake and effectiveness in relation to objectives. Governments and policy-makers require educating on the roles, function and importance of youth involvement in policy formulation, implementation and monitoring and evaluation. Youth advocate groups should be established, and members trained to contribute towards this function. This should be done with the support of other major stakeholders in youth health. However, given the well-recognised inclination of organisations to monitor only those things that are easy to measure, it is important to identify and prioritise monitoring of those elements of the policies and programs that are most meaningful to policy-makers and their target populations.

 Future policy development should include consideration of a combination of feasible and effective approaches such as ASRH information and education in schools, communities and media. Other important issues that can increase policy effectiveness include enhancing access to sexual and reproductive health services by removing cost-related barriers and supporting health workers to provide counselling services. The wider policy environment can also be mobilised to encompass responses to the social determinants of adolescent pregnancy. Finally, qualitative research is required to understand the barriers that impede implementation of policies and programmes.

## Ethical issues

 Not applicable.

## Competing interests

 Authors declare that they have no competing interests.

## Authors’ contributions

 Conception and design: BOA, MK, LP, and FB. Acquisition of data: BOA and MK. Analysis and interpretation of data: BOA, MK, LP, and FB. Drafting of the manuscript: BOA, MK, LP, and FB. Critical revision of the manuscript for important intellectual content: BOA, MK, LP, and FB.

## Disclaimer

 The authors declare that the views expressed in this manuscript are their own and not an official position of any institution or funder.

## Authors’ affiliations

^1 ^School of Public Health, University of Technology Sydney, Ultimo, NSW, Australia. ^2^Faculty of Health, University of Technology Sydney, Ultimo, NSW, Australia.
